# Obesity and Pancreatic Cysts in African American Patients

**DOI:** 10.7759/cureus.3160

**Published:** 2018-08-20

**Authors:** Anahita Shahnazi, Dilhana Badurdeen, Adeyinka O Laiyemo, Mehdi Nouraie, Hassan Brim, Priscilla Wessly, Sahar Geramfard, Ali Afsari, Niel Page, Hassan Ashktorab

**Affiliations:** 1 Department of Medicine, Howard University Hospital, Washington DC, USA; 2 Department of Medicine & Cancer Center, Howard University College of Medicine, Washington DC, USA; 3 Department of Medicine, University of Pittsburgh Medical Center, Pittsburgh, USA; 4 Department of Pathology, Howard University College of Medicine, Washington DC, USA; 5 Pathology, Howard University, Washington DC, USA

**Keywords:** pancreatic cyst, pseudocyst, obesity

## Abstract

Objective

Obesity is one of the risk factors for pancreatic cancer and a prognostic factor for acute-chronic pancreatitis.

Aim

To explore the relationship and association between obesity and pancreatic cysts over a 25-year period in African American patients.

Methods

We reviewed the medical records of 207 patients diagnosed with pancreatic cysts via radiology and pathology data from January 1988 to December 2012. A control group was selected from a separate group of healthy patients without a history of pancreatic disease. The patients were evaluated in five groups according to the last 20 years of diagnosis in five-year intervals.

Results

Most patients with pancreatic cyst (73%) were overweight (defined as a body mass index (BMI) ≥ 25), and 53% had a history of chronic pancreatitis compared to patients in the control group. There was a significant difference between the two groups; 79% of patients group were overweight (BMI ≥ 25) vs. 66% in control group (p = 0.02). The incidence of obese and overweight patients was significant (85%) during the 2008 to 2012 interval for the test group (p = 0.009).

Conclusion

Given the increasing proportion of obese pancreatic cyst patients in recent decades compared to the proportion noted in the 1990s, obesity plays a large role in the formation of pancreatic cysts.

## Introduction

Obesity is one of the risk factors of pancreatic cancer and is a prognostic factor for acute and chronic pancreatitis [1,2]. Pancreatic pseudocysts of the pancreas result from pancreatic inflammation and necrosis. They comprise 15% to 30% of pancreatic cysts overall [1,3] and close to 50% of pancreatic cysts in patients with a history of chronic pancreatitis [4]. There are some risk factors associated with pancreatic cysts and obesity, and their mechanisms have been studied [5]. Recently, visceral fat and adipocytokines were shown to play very important roles in a variety of conditions, including inflammation [5-9]. Obesity can cause inflammation and fatty necrosis that can lead to pancreatic pseudocyst production [6].

The role of obesity and its association with the development of pancreatic cysts has not been well evaluated, especially in African American (AA) patients, and current data are conflicting. Therefore, we aimed to determine the frequency of obesity in African American patients with pancreatic cysts.

## Materials and methods

In this retrospective study, we reviewed the medical records of 207 referred African American patients with chronic abdominal pain, nausea and vomiting, early satiety, and weight loss during the period from January 1988 to December 2012 who were also diagnosed with pancreatic pseudocysts by some attending gastroenterologist at Howard University Hospital based on radiological, clinical, laboratory, and pathology data. In this study, pancreatic pseudocysts were mainly detected by abdominal computed tomography (CT) scans. Cyst had characteristic features such as a well-defined wall, homogeneous fluid density, and no non-liquid components. CT scan with intravenous contrast medium and pathology reports helped to diagnose pancreatic cystic neoplasms. The study was approved by Howard University Institutional Review Board.

We used frequency and median (interquartile range) to show the distribution of categorical variables and age. We used the Kruskal-Wallis or Chi-square test to determine the association between clinical variables and pancreatic cystic neoplasms or chronic pancreatitis. Statistical analysis was done by STATA 13.0 (StataCorp., College Station, TX).

## Results

Pancreatic cyst in African American patients comprised the case group, and a control group was selected from healthy African American patients without a history of pancreatic disease and had normal results on a colonoscopy screening.

The median ages for the case group and control group were 51 years (range, 45 to 59 years) and 57 years (range, 51 to 63 years). Sixty-two percent of patients in the case group were men, and 46% of patients in the control group were men. In the case group, 0.9% of the patient group had pancreatic cystic neoplasms, and the others had pseudocysts. There was a significant difference between the two groups; 79% of the case group were overweight (Body mass index (BMI) ≥ 25) vs. 66% in the control group (p = 0.02). Also, 20% of patients in the case group were obese with a BMI > 30.

Most of the patients (55%, 114/207) with pancreatic pseudocyst had a history of pancreatitis. The control group had significantly more patients who smoke (79% in the control group vs. 66% in the case group (p = 0.02)), but alcohol consumption was not significantly different between the two groups (Table 1).

**Table 1:** Distribution of age, gender, body mass index (BMI), etc. in case vs. control group with p-values.

	Healthy controls N = 140	Patients N = 207	p-value
Age, median (interquartile)	57 (51-63)	51 (45-59)	<0.001
BMI ≥ 25	92 (66%)	163 (79%)	0.02
Male, N (%)	64 (46)	129 (62)	0.002
Chronic pancreatitis, N (%)	0	114 (55)	NA
Diabetes, N (%)	18 (13)	40 (19)	0.1*
Tobacco, N (%)	110 (79)	136 (66)	0.020*
Alcohol, N (%)	84 (60)	120 (58)	0.7*
Gall stone, N (%)	0	13 (6)	NA
Tumor, N (%)	0	18 (9)	NA
* Age and gender adjusted. NA: not applicable comparison because of selection criteria			

In pancreatic cyst patients, 85% of older patients (≥65 years old) and 70% of patients < 65 years had BMI ≥ 25 (p = 0.06). There was no significant difference between patients with pseudocysts and pancreatic cystic neoplasms with regard to demographic or clinical history.

The patients with chronic pancreatitis were significantly younger than patients without chronic pancreatitis (49 vs. 54 years old), tended to be men (70% vs. 53%), were less likely to have diabetes mellitus (11% vs. 29%), and more likely to have gallstones (10% vs. 2%) (Table 2).

**Table 2:** Distribution of demographic and clinical variables by history of chronic pancreatitis.

	No pancreatitis N = 93	Pancreatitis N = 114	p-value
Age, median (interquartile)	54 (47-65)	49 (44-56)	0.002
Body Mass Index (BMI)	68 (73%)	83 (72%)	0.9
Male, N (%)	49 (53%)	80 (70%)	0.01
Diabetes, N (%)	27 (29%)	13 (11%)	0.001
Tobacco, N (%)	30 (30%)	41 (36%)	0.6
Alcohol, N (%)	34 (37%)	53 (46%)	0.2
Gall stone, N (%)	2 (2%)	11 (10%)	0.03

**Figure 1:**
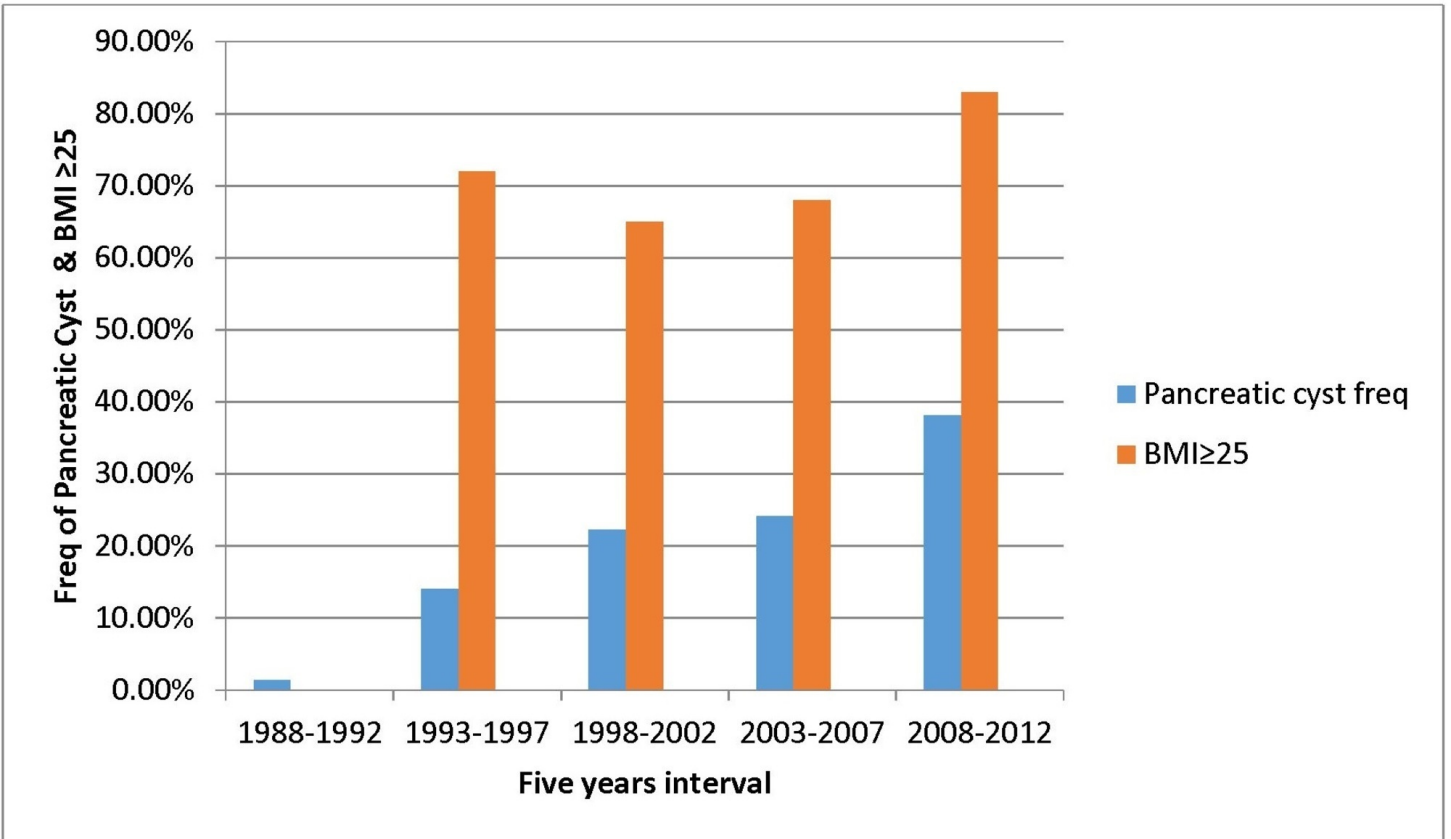
Pancreatic cyst frequency and body mass index (BMI) according to the year of diagnosis in five years interval.

Finally, the patients were evaluated in five groups according to the year of diagnosis in the five-year interval. For the first group (those diagnosed before 1997), we had no overweight patients. The incidence of being overweight among the case group was 83% during the last five-year interval (2008 to 2012, p = 0.009). The rate of being overweight (BMI ≥ 25) from 1993 to 2012 has increased from 72% to 83%, respectively. The frequency rate of pancreatic cysts among our patients has increased from 1.4% to 38.1% (Figure 1).

## Discussion

This study evaluated the incidence of obesity-overweight among African American patients with pancreatic cysts and demonstrated that the incidence of obesity has increased among patients with pancreatic cysts from 2008 to 2012. Our study showed a significant BMI difference between the two groups. However, the incidence of being overweight (BMI ≥ 25) was not the same in different five-year intervals. During the interval from 1993 to 2012, the incidence of overweight status increased. Pancreatic cyst patients had the higher incidence of being overweight from 2008 to 2012.

We had some limitations for selecting the control group in this study. The rate of obesity in the general population of African Americans published by the Centers for Disease Control and Prevention increased from 54% in 1994 to 69% in 2012 [10]. It demonstrates the rate of obesity among patients with pancreatic cysts is higher than that of the general population and assumes obesity is a risk factor for the development of pancreatic cysts even without a history of chronic pancreatitis. However, to prove this hypothesis, we require an additional study with a large number of patients in the test and control groups gathered from more recent years. In elderly patients (i.e., those aged ≥ 65 years), obesity’s effect as a risk factor to develop pancreatic cysts is greater than in other patient age groups. In our pancreatic cyst patients, 85% of patients ≥ 65 years had BMI ≥ 25, which was more than the incidence of obesity in the general African American population.

Although pseudocysts and cystic tumors have different pathophysiology, obesity can play a big role in cyst production and development. Pancreatic cancers are more likely to exist in men than in women and more in African Americans than in whites [11,12]. Smoking increases one's risk of pancreatic cancer two to three folds. Alcohol consumption is also considered a risk factor for pancreatic cancer [13-19]. In acute and chronic pancreatitis, obesity is a significant prognostic factor during hospitalization, and BMI independently predicts complications [20,21].

Visceral adipose tissue (VAT) volume is strongly correlated with the formation of a pseudocyst and with systemic inflammatory response syndrome in patients with acute-chronic pancreatitis. Indeed, high VAT volume may lead to severe acute-chronic pancreatitis [7,22].

Obesity in the United States has been increasingly cited as a major health issue in recent decades [23], and our study shows that the rate of pancreatic cysts and its association with obesity is also increasing in recent decades. Currently, the detection rate of pancreatic cysts has increased due to the utilization of imaging facilities. However, the rate of obesity has increased in the United States, too. Therefore, it is difficult to disregard the role of obesity in pancreatic cyst formation because of the concurrent increase in the prevalence of obesity.

## Conclusions

Given the increasing proportion of obese pancreatic cyst patients in recent decades compared to the proportion noted in the 1990s, obesity plays a large role in the formation of pancreatic cysts.
